# Politics and its intersection with coverage with evidence development: a qualitative analysis from expert interviews

**DOI:** 10.1186/1472-6963-13-88

**Published:** 2013-03-09

**Authors:** Danielle Bishop, Joel Lexchin

**Affiliations:** 1 Graduate Program in Health, York University, Toronto, Canada; 2 School of Health Policy and Management, York University, Toronto, Canada; 3 Emergency Department, University Health Network, Toronto, Canada; 4 Department of Family and Community Medicine, University of Toronto, Toronto, Canada

**Keywords:** Australia, Canada, Coverage with evidence development, Governance, Key informants, Pharmaceuticals, Politics, United Kingdom, United States

## Abstract

**Background:**

Pressures on health care budgets have led policy makers to discuss how to balance the provision of costly technologies to populations in need and making coverage decisions under uncertainty. Coverage with evidence development (CED) is being employed to meet these challenges.

**Methods:**

Twenty-four interviews were carried out between June 2009 and December 2010 with researchers, decision makers and policy makers from Australia, Canada, United Kingdom and United States. Three phases of coding occurred, the first being manual coding where the interviews were read and notes were taken and nodes were extracted and imputed. NVIVO coding was applied to the interview transcripts, with both broad general searches for word usages and imputed nodes.

**Results:**

Four overarching thematic areas emerged out of contextual analysis of the interviews – (1) what constitutes CED; (2) the lack of a systematic approach/governance structure; (3) the role of the pharmaceutical industry and overt political considerations in CED; and (4) alternatives and barriers to CED. We explore these themes and then use concrete examples of CED projects in each of the four countries to illustrate the political issues that our interviewees raised.

**Conclusion:**

Until the underlying political nature of CED is recognized then fundamental questions about its usefulness and operation will remain unresolved.

## Background

In 1848, Rudolph Virchow stated that, “medicine is a social science, and politics is nothing else but medicine on a large scale” [1]. We are currently bearing witness to an inverse example of Virchow’s reasoning in a new scheme, aptly called coverage with evidence development (CED) or access with evidence development (AED) (the two terms are used interchangeably). Pressures on health care budgets have led policy makers and key stakeholders to move into discussions around how to balance the provision of costly innovative technologies to populations in need with making coverage decisions under uncertainty. This is where CED enters the debate, representing an instance whereby medicine is nothing else than politics on a large scale. Here we are using the term “politics” to mean the interests that the various stakeholders (industry, government, patient groups, professional associations) bring to the table and how they intersect in coming to a final decision. These interests are in evidence when stakeholders are negotiating both about whether or not to undertake a CED study and the conditions under which a particular product will be funded.

CED is an umbrella term for institutions and policy think tanks to capture the essence of various evidence development approaches. It is a mechanism for going beyond a binary yes/no decision about coverage for new technologies or drugs by offering coverage in the context of prospective studies [2]. It provides an alternative “in situations in which the technology [or drug] does not appear to meet the standard criteria for reimbursement, predominantly because of uncertainty surrounding the existing evidence base and when additional data collection could reduce this uncertainty” [3].

According to Menon et al. [4], variations on the approach are placed at times under the auspices of terms such as ‘only in research,’ ‘pragmatic trials,’ ‘comparative effectiveness research,’ ‘field evaluation,’ ‘policy trials,’ and ‘real time monitoring’. As Hutton and colleagues claim, “Coverage with Evidence Development is one of several policy options that have been posited to overcome problems associated with making coverage decisions under uncertainty” [5]. Indeed, though not entirely new or novel, CED is the approach that has attracted the most attention from policy makers and is also the one with the largest amount of literature.

In over fifteen years of research on CED since 1995, the study design, desired outcomes, and the application of conditions or agreement on a particular conditionality remain neither straightforward nor standardized [6]. In general, CED is understood to comprise two different typologies. The first is where “payers provided interim funding for a technology … to be used in a clinical study intended to collect information needed to reduce decision-making uncertainties (i.e. coverage as part of a clinical study)” [7]. The second are those cases “based on some form of outcomes guarantee implemented through contractual arrangements between payers and manufacturers (i.e. coverage with outcomes guarantee)” [7]. In this paper we focus only on the former definition, i.e., defining CED as a process that uses data from provisionally covered populations to determine whether to continue coverage, expand or contract it and under what conditions.

CED is often presented as a seemingly neutral alternative or antidote to the challenge of funding under conditions of uncertainty. Its delivery mechanisms indicate much promise for achieving the mandate of simultaneously allowing access to more diffusive health technologies whilst maintaining evaluative mechanisms to assess their benefit and efficacy. “CED permits provisional coverage of selected treatments lacking adequate evidence of benefits and risks in the context of planned research to develop the evidence needed to determine whether definitive coverage is warranted” [6]. In the end, decisions about how to spend scarce resources need to be made and, regardless of the methods used to push ahead, in circumstances of decision-making and defining a legitimate scope, power and politics always intermingle with evidence. Though it is a promising alternative, broaching a policy for CED is an inherently political process whereby “the legitimate scope of CED, however, remains poorly defined” [6].

The interviews on which this study is based emerged out of an attempt to further understand how various stakeholders perceive both the potential and the challenges of CED with a view to gauging what is needed when moving forward, such as processes required to help define the legitimate scope of CED and to build the governance structures of CED. Interviews allow us to explore the complexities of CED in more depth than could be achieved just through documentary review or a study of the currently published literature. The literature on CED has talked about it as a political process, but this level of discussion tends to be peripheral to its other aspects.

The aim in undertaking the analysis of the interviews is to take the debate about CED in a novel direction by examining its tensions and complexities in light of the exercise of power relations, asking how these shape what becomes knowledge and how that knowledge is interpreted into the decisions that result from CED schemes. Specifically we are interested in CED as it relates to decisions about pharmaceuticals. We dissect the interviews to extract selected themes that we think allows for a fuller understanding of the political undercurrents in the decision-making process, and the relations of power that determine how and under what circumstances CED is used. We then link these themes to CED projects that have been undertaken to illustrate its essential political nature.

## Methods

The British Columbia Coverage with Evidence Development (CED) project is a two-year research endeavor receiving funds from the College of Pharmacists of British Columbia. The purpose of the project is to gain an understanding of the barriers associated with planning, implementing and evaluating CED-related research. In addition, the project intends to describe and investigate the unresolved policy issues around covering pharmaceuticals with evidence development.

This portion of the research project involved interviews with a variety of stakeholders (researchers, policy makers and decision makers). These actors were deliberately chosen as their perspectives would allow for an investigation of the spectrum of policy issues associated with CED-type research and generate key “lessons learned” from international and Canadian experience with CED. Researchers, typically based in academic institutions, were defined as those who were concretely involved in the design, conduct and evaluation of studies on CED or the management of data used in CED studies. Policy makers were those who provided advice about how CED should be used in the decision-making process and finally decision makers were those who worked in government funded agencies and who took the evidence generated from the studies to make a final decision about funding the drug or technology.

Interview subjects were initially identified through enquiries with members of the International AED working group, and subsequent interviewees were identified through snowball sampling and internet searches. We chose candidates for interviews based on a reading of the literature on CED and to reflect the countries that had the most experience in the area [7,8]. We restricted our choices to English speaking countries due to language limitations among the people conducting the interviews. The interviewees were involved in CED for technology, e.g., evaluation of positron emission tomography scanning and implantable cardiac defibrillators, for surgical procedures, e.g., lung reduction surgery, and for pharmaceuticals. We used a semi-structured interview guide with common questions for all categories of interviewees and then specific questions depending on their specific role. Open-ended questions allowed interviewees to elaborate on particular areas that they regarded as important. A total of six different individuals conducted interviews, with most interviews conducted by two (AC and RH).

A total of 24 interviews (approximately 45-minutes each) took place between June 2009 and December 2010. Most of these interviews were conducted by phone and the resulting interview notes were analyzed to identify themes and policy issues. With permission, some of the interviews were recorded for the purpose of post-interview note taking. We used a grounded theory approach to extract themes from the interviews [9]. Interview transcripts were first manually coded and entered into NVIVO data analysis (version 9) through identification of nodes and themes. Three phases of coding occurred, the first being manual coding where the interviews were read and notes were taken and nodes were extracted and imputed. NVIVO coding was applied to the interview transcripts, with both broad general searches for word usages and imputed nodes. This method generated varied themes and sub-thematic areas that captured the essence of the debate. A single author (DB) initially coded the interviews and the second author (JL) subsequently independently read and coded them to ensure that all key themes were captured. Interviews were iteratively read until thematic saturation was reached. Any differences were resolved through discussion until consensus was reached.

Interviewees were promised anonymity in any publication and as a result we only identify them by their role in the process and the country where they work, i.e., researcher, decision maker or policy maker and Australia, Canada, United Kingdom (UK) or United States (US). Any other parts of the interview that might identify them, e.g., specific place of work, the name of drug or device has also been removed. The sponsor played no role in the design of the questionnaire, the conduct of the interviews, their analysis or the writing of the manuscript. Ethics approval was received from the Human Participants Review Committee at York University.

## Results and discussion

Of the 24 individuals who were interviewed 16 were researchers, 5 decision makers and 3 policy makers. These individuals came from Australia (4), Canada (11), UK (7) US (2). Interviewees from Australia, the UK and the US were almost exclusively working in the area of pharmaceuticals except for one from the UK and one from Australia, who concentrated on technology and procedures, respectively. The plurality (4) of the Canadians worked in the drug area, 3 worked in technology, 3 in drugs and technology and 1 worked on both drugs and procedures.

Although interviewees were involved in CED in different areas and spoke about their different experiences we consciously selected observations and opinions that could be applied to our focus on pharmaceuticals.

Four overarching thematic areas emerged out of contextual analysis of the interviews – (1) what constitutes CED; (2) the lack of a systematic approach/governance structure; (3) the role of the pharmaceutical industry and overt political considerations in CED; and (4) alternatives and barriers to CED. In identifying and the explaining these themes we use selective quotations from the interviewees to help illustrate the points that they are making and to allow them to express their opinions in their own words.

The following sections expand on these areas by identifying subthemes contained in each broad theme. (See Table 1 for a summary of the key points.) It should be noted that although these themes are presented as separate they are all interrelated, reflecting the complexity of CED.

**Table 1:** Key points from the interviews

**Theme**	**Subtheme**	**Key points**
**What constitutes CED**	*What is worthy of research*	• Conditionality is most important, e.g., drug or device, characteristic of disease
	*What constitutes uncertainty and how should it be measured*	• Determining uncertainty is an art
	*What constitutes evidence and how should it be managed*	• How much agreement should there be on minimum evidence expectations
		• CED can become a never ending series of studies
**Lack of a systematic approach/governance structure**	*Role of different stakeholders*	• Where should leadership rest
		• Over involvement of multiple stakeholders
		• Engage disease advocacy groups
	*Translation of research into policy*	• Requires guidelines and a discontinuation policy
		• Limited data and financial considerations are barriers
		• Justifying the continued flow of funds requires policy standardization and a formal agreement of the strategy to be put in place
**Corporate influence and overt politics in CED**	*The political process as part of CED*	• Political processes in terms of the source of money and decision-making are at a level above researchers
		• Political processes can undermine the ability to achieve real change
		• Researchers blame policy makers and decision makers for problems in translating research into policy
	*The role of the pharmaceutical industry*	• Researchers, policy makers and decision makers are all skeptical about the role of industry
**Alternatives and barriers to CED**	*Alternatives*	• Preference expressed for risk-sharing and tax breaks over CED
	*Barriers – data and privacy issues*	• Concern that decision makers are not willing to be accountable
		• Doctors not cooperative enough
		• Need better access to data and the ability to link databases
		• Registries useful but come with their own set of problems

### What constitutes CED?

Perceptions of CED differ widely between the interviewees according to their roles and involvement in research, decision-making, and policy development. Interestingly, interviewees from all three categories seemingly suggest agreement on what CED is not, such as “Evaluations of products that are already on the market, like insulin pumps in Ontario, are not CED” (Canada Researcher 1). The debate largely revolves around how to constitute what it actually is, reflecting a grey area in which differing descriptions of what distinguishes studies that are CED, and those that are not, emerge to create some tension amongst stakeholders. For instance, one policy maker observed that although his group did safety studies of already marketed drugs he questioned if this fit the definition of CED (Canada Policy Maker 1). A researcher felt the same way stating “evaluations of products that are already on the market…are not CED” (Canada Researcher 1).

Given the general feeling that CED should not deal with existing drugs and devices, three lingering questions revolve around sub-debates. First, the degree of (or nature of) uncertainty needed to move ahead with this approach; second, the conditions that would allow the approach to unfold; and third, the deliberations driving the evidence base that is, at the end, the point of introducing the innovation. As one interviewee put it:

“Some people see it as a way to bypass the regular standards and others see it as an easy way to get something in that they’ll never get out…It is less about evidence and about the system” (Canada Researcher 5).

Although one decision maker called CED a policy tool (Canada Decision Maker 1), another termed it a research tool (UK Decision Maker 1), while a researcher was more practical in calling it an evaluation tool (Canada Research 1). At the core of the discussion are differing perceptions regarding both the definitional and methodological issues and, as well, the scope of CED. We further dissect these facets of debate within three interrelated themes that illustrate the boundary issues: what is worthy of research, what constitutes uncertainty and what constitutes evidence?

i. What is worthy of research?

“Is putting off a decision a reason to use CED? Is that an incentive?” While one interviewee answered ambiguously but without hesitation by stating “Probably” (Canada Researcher 4), another believed that circumstances alone defined what is and is not worthy of CED, e.g., what is the character of the disease and the population that it affects (UK Policy Maker 1). In short, the challenge in moving forward is ascertaining the conditionality. For instance, when an institutional report states “Yes” to a particular drug or device it is usually done so at the discretion of adding a disclaimer: “but, with a certain number of conditions.” However, these conditions are not necessarily consistent – they may be made on an ad-hoc basis (Canada Decision Maker 1), or they may depend on whether the decision involves a health device or a drug (Canada Researcher 1).

ii. What constitutes uncertainty and how should it be measured?

On the one hand CED is considered a way of managing uncertainty –“that’s all that it is” (Australia Decision Maker 1). But what actually constitutes uncertainty is not straightforward as it is inherently linked to issues of risk, safety and evaluation. As one interviewee eloquently said, “There are rules for creating evidence but when push comes to shove, it’s an art [evaluating evidence]” (US Decision Maker 1).

iii. What constitutes evidence and how should it be managed?

At the crux of the dilemma of defining uncertainty is the evidence on the efficacy, effectiveness and safety of new technologies. Evidence-based research must begin with questions, and this is part of the debate regarding CED; can people agree on the minimum evidence expectations and do those concerns include just effectiveness or, in addition, safety?

“The message that came across early was that the decision makers have to drive this…[They] need to decide what they want to know” (Canada Researcher 5).

“When we decide on CED we have to be fairly certain about safety” (US Decision Maker 1).

There is also the worry that CED can become a never-ending series of studies. How much evidence is enough? For instance, if CED is begun on one product and before this research is completed a second drug for the same problem is marketed, should the CED be abandoned or extended to include the newer product (Australia Researcher 1)?

### Lack of a systematic approach/governance structure

CED lacks a governance structure, or a systematic approach. As one interviewee noted, “schemes are malleable post implementation…[it has] a controversial typology and is ad hoc” (UK Researcher 4). Another mentioned that it is ad hoc and “needs structure and protocol” or “a decision maker’s field guide or a tool kit” (Canada Researcher 5). Beyond ascertaining uncertainty, three aspects of governance or approach to CED act as barriers and contribute to the difficulty in establishing norms.

i. Role of different stakeholders

With respect to CED, researchers took contradictory positions around where the leadership should rest. Although one opined that it would be better for clinicians to be in a leadership position with respect to CED, rather than leaving it to government (Australia Researcher 3), another noted that clinicians, i.e., researchers, were more interested in using the technology than in generating evidence about it (UK Researcher 5). A third conflicting opinion maintained that researchers can contribute to developing the methodology around CED but it is up to “decision makers to decide what they want to know. It means that you are getting them to ask the right questions” (Canada Researcher 5). But, what are the right questions? A fourth felt that there was over involvement of multiple stakeholders in running CED studies; this interviewee identified a wide variety of organizations and groups including the pharmaceutical industry, the department of health, patient groups and clinicians (UK Researcher 2). Finally, a UK decision maker singled out the need to also engage disease advocacy groups so as to be able to demonstrate to them that their involvement was sought (UK Decision Maker 1).

ii. Translation of research into policy

The role of different stakeholders is inherently linked to the difficulty in translating research into policy or implementing policy. This process requires guidelines and a firm grounding in not only what constitutes evidence or efficacy but, also as one interviewee held, “in order to look into effectiveness or efficacy, we need a discontinuation policy because we don’t know the benefit [before the drug is tested]” (Australia Decision Maker 1).

Limited data and financial considerations are also barriers.

“If the research leads to a decision that a new technology should be implemented, but the budget is not available for implementation, this is a barrier” (Canada Researcher 1).

“The rules of the game need to be set up front…There has to be an explicit understanding, the public and everyone else, that if it doesn’t perform you are going to pull the money” (Canada Researcher 3).

Justifying the continued flow of funds requires policy standardization and a formal agreement of the strategy to be put in place; currently it is a cyclical schema that has a lot of holes. Indeed, making conscious and collective decisions around evaluation and cost and measuring certainty are highly difficult. The politics and history undergirding the lack of agreement acts as a strong barrier to doing so and impacts the translation of CED.

“There’s nothing more complicated than making a decision…We have decades of bad behavior of making decisions [by the Ministry of Health] based on a whim or ad hocing” (Canada Researcher 3).

### Corporate influence and overt politics in CED

The translation of evidence into policy is riddled with political and economic considerations, both the overt political process involved in CED and the role of the pharmaceutical industry.

i. The political process as part of CED

The most explicit evidence of relations of power comes from the hierarchy of roles in the decision-making process. Researchers from both Canada (Canada Researcher 2) and the UK (UK Researcher 1) identified political influences as being above them in terms of both determining where the money for CED will come from and where the ultimate decision-making comes from. This echoes the contentious theme that even though political processes may help researchers get funding for their projects they can also undermine the ability to achieve any real change (Canada Researcher 5). As one researcher identified, there are imbalances and risks inherent in the governance of CED. In some instances agreements make “it possible for parties of the scheme to rewrite the rules subsequently,” which is a dangerous practice that places the resources that the society or health system puts into the scheme at increased risk (UK Researcher 4). Finally, with the difficulties and barriers taken together, researchers tended to blame decision and policy makers for the trouble they encounter in translating the evidence gained through CED into policy. Not only because of the perceived flaws in the research.

(US Researcher 1) but because agreement on decision-making, as opposed to being ‘ad hoc’ is imperative: “once you let something out, you can’t get it back” (UK Researcher 3).

ii. The role of the pharmaceutical industry

Decision makers, policy makers and researchers all voiced some level of criticism about the role of the pharmaceutical industry in CED. A policy maker remarked

“There is one…registry they [the industry] are interested in…It’s also got industry funding – we are trying to work out how to analyze the data and manage the conflicts of interest” (Canada Policy Maker 1).

A researcher felt that “the pharmaceutical industry is trying to get away with things” (UK Researcher 5) and these same sentiments were reflected in what a decision maker said.

“It’s more the industry-sponsored studies that we have to vet carefully” (US Decision Maker 1).

### Alternatives and barriers to CED

i. Alternatives

One Australian decision maker felt that there were better approaches to managing uncertainties than using CED approaches. This person was in favor of using risk-sharing because it took the responsibility from the payer and shared it with the sponsor. This decision maker would rather “get the structures right, so we can interrogate data as is appropriate and have everyone have confidence in that interrogation” (Australia Decision Maker 1). Another Australian, in this case a researcher, also talked favorably about risk-sharing by identifying a disadvantage of CED. With CED, this person stated, there “is the potential of investing [in] technology that might later not be cost-effective [or] proved to have safety issues” (Australia Researcher 2). These preferences for an alternative to CED from Australian informants may reflect the relative successes of the long-standing use of pharmacoeconomic evaluations by the Pharmaceutical Benefits Scheme (PBS) [10]. Meanwhile, a UK researcher advocated an entirely different approach and held a different rationale for his views.

“So I would do things like give bigger tax breaks to R&D process[es] that follow a rational analysis and set a high threshold for an expected health gain in order to [get] something from Phase 2 to Phase 3 [drug trials]” (UK Researcher 4).

ii. Barriers – data and privacy issues

People from different backgrounds laid the blame for problems with CED on different target groups. One researcher was critical of decision makers.

“There is basically a general commitment to appearing to be accountable that is primarily financial and media – don’t look stupid and don’t spend money that you shouldn’t be spending” (Canada Researcher 6).

On-the-other hand, a decision maker laid much of the blame for the difficulties in doing CED on physicians’ problems in collecting data and asking people to sign informed consent forms (Canada Decision Maker 2). There was also a structural issue that was identified, namely the access to data and the privacy issues that this entails. There was a general agreement that, in order to successfully undertake CED, it is necessary to have better access to data and to be able to link databases. We “need info as an evaluation tool” (Canada Researcher 1). “We are looking at standardizing data elements across all our registries and they are all aimed at being in line with the national health data dictionary” (Australia Researcher 3). Thus, registries were identified as one key method of collecting the needed data by all categories of interviewees (Canada Policy Maker 2, Canada Researchers 3 & 4, US Decision Maker 1). However, with registries comes the problem of finding a sustainable funding model for them, verifying the accuracy, reliability and completeness of the information (Australia Researcher 3) and, as well, ensuring the privacy of the information that is stored (Canada Policy Maker 1, Canada Researcher 5).

Previous authors have identified a number of unresolved issues with CED. Hutton and colleagues discussed the dominant logistical advantages and disadvantages of CED. Making decisions about coverage too early in the lifecycle of a product may lead to paying for clinically or cost-ineffective products; waiting too long may harm patients who could have benefited from the product [5]. Lexchin [8] focused on the problem of limited data for pharmaceuticals as the reason for undertaking CED. There are also inherent ethical challenges to CED approaches that were summarized in the proceedings from a symposium held in Banff, Alberta in February 2009. As one example, once a decision has been made to fund a drug or device under CED, regardless of the evidence, it becomes exceptionally difficult to discontinue the funding [11].

All of these questions are inherently political in nature but none have been approached from a primary political perspective. We take as our trajectory the sentiment of one interviewee: “…it is less about evidence and [more] about the system” (Canada Researcher 5). We recognize that our interviewees come from multiple backgrounds and that the CED process in the four countries may have national differences, e.g., who funds the studies, what level of government has the decision making power. Yet, we also believe that the political tensions, reflected in the interests of the different parties, undergirding CED are common to all countries and that these commonalities ultimately transcend any of the national variations. It is the political tensions that are the focus of our analysis. In undertaking this analysis we draw on examples of CED projects from various countries, not in an attempt to criticize the process or any particular group, but to ground and further illustrate the themes that have emerged from our interviews.

All knowledge, once applied in the real world, has effects, and in that sense at least, ‘becomes true.’ Knowledge, once used to regulate the conduct of others, entails constraint, regulation and the disciplining of practice. Thus, there is no power relation without the correlative constitution of a field of knowledge, nor any knowledge that does not presuppose and constitute at the same time, power relations [12].

Our interviewees identified two key tensions, the first being how the various stakeholders can affect political decisions around initiating a CED project and, the second, the influence of the pharmaceutical industry in CED. These issues are illustrated in the CED project that the British Columbia (BC) government initiated into drugs for Alzheimer’s Disease. The Alzheimer‘s Drug Therapy Initiative (ADTI), begun in 2007, was designed to provide data on the safety, effectiveness and appropriate use of the cholinesterase inhibitor class of drugs in the treatment of dementia and to inform government policy on the coverage of these drugs [13]. People intimately involved with the ADTI acknowledged that the project would never have been started without direct and indirect pressure from the pharmaceutical industry on government, physicians, Alzheimer’s Disease interests groups, and Alzheimer’s patients and their families. The provincial government had previously rejected funding for this group of drugs, yet was undergoing pressure to cover them, BC being the last province in the country to do so. Other people from the ADTI reinforced the observation by our interviewees that once the BC government started to pay for these drugs it would be very hard to reverse its decision regardless of the evidence around effectiveness. Although money for the CED project comes entirely from the Ministry of Health, the companies making the 3 drugs being studied provide the Ministry with funds equal to the cost of the first three months therapy of their drug for each patient. The companies are also funding educational sessions for doctors on the proper diagnosis and treatment of Alzheimer’s Disease and are assisting with patient recruitment. Thus, the close working relationship amongst the industry, the physicians and the consumer groups connected to the ADTI has caused some, but not all people, to question how much the integrity of scientific research and the academic freedom of individual researchers are or will be affected by these ties (Alan Cassels, personal communication).

The question of the involvement of the pharmaceutical industry is also illustrated in an Australian study looking at bosentan, a drug for the treatment of pulmonary arterial hypertension (PAH). Eight of nine authors of the main outcome study had a significant relationship with Actelion Pharmaceuticals, the maker of the drug [14]. While there is no evidence that this level of COI biased the findings of the study it still raises concerns given previous research into the association between author COI and the outcome of clinical research [15,16]. The bosentan study involved the creation of a patient registry and raises the political issues inherent in registries – who will govern the registry, how will privacy be safeguarded, who will have access to the data and where will the funding to maintain a registry come from [17].

Australian and British interviewees raised the question about whether CED was actually the best approach to take in getting new and cost-effective drugs funded. Australia required bosentan to undergo a CED evaluation in order to be funded under its PBS. But, since bosentan was listed, at least 4 other treatments for PAH have also been listed on the PBS without a CED requirement. According to a meta-analysis, some of the more recent products appear to offer mortality benefits that bosentan doesn’t [18]. This outcome raises a number of issues – was the time and the money for the bosentan CED wasted, why were the other products listed without undergoing CED, why is bosentan still being listed if other products are superior? While no one has written about these decisions from a political perspective, it seems reasonable to expect that there are political undertones to the answers to some of these questions.

A number of informants identified questions as to the purpose of CED, the role of different stakeholders in the governance of a CED project, and the process for funding a study. These issues come to the fore in the Canadian Fabry Disease Initiative that was initially conceived of as a 10-year study of enzyme replacement where two different versions of the enzyme were available for people with a deficiency of alpha galactosidase. The project itself appears to have been initiated at least in part as a result of patients with Fabry Disease lobbying for government payment for a drug that can cost C$300,000 annually per person [19]. Funding for the project came from the two companies making the enzyme, the provincial and territorial governments and Health Canada, the latter of which agreed to fund the study for three years with the money administered through the Canadian Institutes for Health Research (CIHR). At the end of the 3 years Health Canada announced that it was terminating its share of the funding – putting the entire project into question. CIHR additionally refused to become the study sponsor because it was not the funder [20]. Access to the confidential 3-year funding agreement has been withheld even from the researchers. Moreover, there is even a dispute between the Canadian Organization for Rare Diseases (CORD), the group representing the patients, and Health Canada as to the reason for the study. CORD claims that the study is being done only because governments refused to fund an expensive therapy while Health Canada maintains that its purpose is to learn more about the effects of the drugs and “to better understand the research challenges associated with drugs that treat small populations” [19]. Investigators have been unable to amend the trial protocol because any changes have to be approved by all provinces providing funding, the two companies and the research ethics boards at each of the nine study sites [21].

Questions posed around evidence, such as how much evidence is enough and how does the evidence affect decision-making, all are raised by the problems identified in the multiple sclerosis (MS) risk sharing scheme running in the UK since 2002. At the end of 2009 the first report from the scheme appeared documenting the status of patients during 2005–7. The patients on therapy fared far worse than those on placebo. This should have triggered a reduction in the price being paid for the two drugs being examined according to the trial agreement. Instead the report claimed that “the scientific advisory group considered that it was premature at this stage to reach any decision about re-pricing the drugs without further follow-up and analyses” [22]. The two companies making the different drugs were part of the advisory committee that recommended not changing the price. This decision, although defended by some [23], highlights the point about how evidence can be used, or misused to the (dis)advantage of some. A team from the Sheffield School of Health and Related Research was originally designated to monitor patient outcomes but later withdrew from its role over concerns about governance of the project and the arrangements for data access and publication rights [22]. As of June 2010 there have been no further annual reports published on the scheme, again reinforcing the point around the control of evidence. Finally, the MS scheme makes the point that evidence comes at a cost and that spending the money is always a political decision. The annual cost is close to £50 million (US$78 million) which Raftery asserts may make it “the most expensive publicly funded ongoing health related study in the UK” [22].

The US CED project that examined the use of high-dose chemotherapy with autologous bone marrow transplant for women with breast cancer is an example of how decision-making around what should be eligible for CED becomes warped and how public pressure can change the nature of studies. Although there was very little evidence that this type of approach actually worked, the high profile of breast cancer, and its often dismal outcome, lead Blue Cross and Blue Shield (BCBS) to offer coverage for the procedure to the 4 million federal employees that it covered. An evidence-based review undertaken by the BCBS Technology Evaluation Center triggered the initiation of a project to evaluate the effectiveness of this new therapy against conventional chemotherapy. The overwhelming public perception was that BCBS was only participating in these trials as a way to avoid paying for the high cost of the treatment. As a result, the federal Office of Personnel Management mandated that the plans could not require coverage in the context of a clinical trial, i.e., that women could receive coverage even if they were not enrolled in the trial. This ruling both undermined the project and reduced enrollment [24].

## Conclusion

Though analyses of CED policies abound, and scholars agree that it is rooted in a health services and research paradigm, they often underemphasize the fact that it operates primarily through political and economic relations. As the above debates reinforce, CED is a *field* of knowledge but one whose boundaries are not yet settled because its political and economic roots remain largely ignored. As a result, tensions emerge in multiple areas such as how evidence should be interpreted, the roles and responsibilities of the various stakeholders and who is responsible for funding decisions. These tensions cannot be abstracted from the political and economic relations that are providing the impetus to develop the field but at the same time, hindering its ability to become the method of choice for resolving the problem of funding in the face of imperfect knowledge. As a result, “the legitimate scope of CED…. remains poorly defined” [6].

Understanding the barriers and potential of a CED framework requires understanding the degree to which it is intrinsically rooted in the fluid and constant exchange of power by virtue of its relationship with government and the other stakeholders who operate within the sphere of CED. Foucault once wrote that, “knowledge, once used to regulate the conduct of others, entails constraint, regulation and the disciplining of practice” [12]. Though the questions posed by our interviewees were explicitly referencing the process of CED, these same sentiments beg questions surrounding the nature of the power-relations that implicitly regulate and constrain stakeholders, decision makers, and researchers. Until the underlying political nature of CED is recognized, the explicit and fundamental questions posed by the interviewees about its usefulness and operation will remain unresolved. To this end we feel that it is necessary to redirect the research into the CED process to not only examine its technical and ethical questions but also its political ones. Future CED studies should be prospectively planned to include an exploration of the political dimensions of the environment in which the study is taking place and to also involve an analysis of the interests of the various stakeholders at various stages of the project.

## Competing interests

Danielle Bishop has no competing interests to report.

In 2007 Joel Lexchin was a consultant to a law firm acting for Apotex Inc. In 2008 he was an expert witness for the Canadian federal government in its defence against a lawsuit challenging the ban on direct-to-consumer advertising. In 2010 he was an expert witness for a law firm representing the family of a plaintiff who allegedly died from an adverse reaction from a product made by Allergan. He is currently on the Management Board of Healthy Skepticism Inc. and is the Chair of the Health Action International – Europe Association Board.

## Authors’ contributions

JL had the initial idea for the project. JL and DB extracted information from the interviews. DB wrote the first draft of the manuscript and JL and DB revised the manuscript. DB and JL approve the manuscript.

## Authors’ information

Danielle Bishop is a PhD candidate in the Graduate Program in Health at York University, Toronto, Canada. Her research interests are global health, the geopolitics of humanitarian aid, and the social and political determinants of maternal-child and reproductive health in protracted refugee settings.

Joel Lexchin is a professor in the School of Health Policy and Management at York University, Toronto, Canada and an emergency physician at the University Health Network. He has published extensively on all aspects of Canadian pharmaceutical policy as well as international pharmaceutical issues. In addition, he has been a consultant to the Canadian federal government, the Ontario government, the World Health Organization, the New Zealand government and the Australian National Prescribing Service.

## Pre-publication history

The pre-publication history for this paper can be accessed here:

http://www.biomedcentral.com/1472-6963/13/88/prepub
